# 4D printing smart biomedical scaffolds with novel soybean oil epoxidized acrylate

**DOI:** 10.1038/srep27226

**Published:** 2016-06-02

**Authors:** Shida Miao, Wei Zhu, Nathan J. Castro, Margaret Nowicki, Xuan Zhou, Haitao Cui, John P. Fisher, Lijie Grace Zhang

**Affiliations:** 1 Department of Mechanical and Aerospace Engineering, The George Washington University, Washington DC 20052, USA; 2 Fischell Department of Bioengineering, University of Maryland, College Park, MD 20742, USA; 3 Department of Biomedical Engineering, The George Washington University, Washington DC 20052, USA; 4 Department of Medicine, The George Washington University, Washington DC 20052, USA

## Abstract

Photocurable, biocompatible liquid resins are highly desired for 3D stereolithography based bioprinting. Here we solidified a novel renewable soybean oil epoxidized acrylate, using a 3D laser printing technique, into smart and highly biocompatible scaffolds capable of supporting growth of multipotent human bone marrow mesenchymal stem cells (hMSCs). Porous scaffolds were readily fabricated by simply adjusting the printer infill density; superficial structures of the polymerized soybean oil epoxidized acrylate were significantly affected by laser frequency and printing speed. Shape memory tests confirmed that the scaffold fixed a temporary shape at −18 °C and fully recovered its original shape at human body temperature (37 °C), which indicated the great potential for 4D printing applications. Cytotoxicity analysis proved that the printed scaffolds had significant higher hMSC adhesion and proliferation than traditional polyethylene glycol diacrylate (PEGDA), and had no statistical difference from poly lactic acid (PLA) and polycaprolactone (PCL). This research is believed to significantly advance the development of biomedical scaffolds with renewable plant oils and advanced 3D fabrication techniques.

Stereolithography is one of the most important solid freeform fabrication techniques for manufacturing constructs with precise geometries designed using computer-aided interfaces^1^. When fabricating 3D scaffolds using stereolithography, photo polymerization of liquid resins is spatially controlled to achieve predesigned structures. However, the commercial availability of liquid resins suitable for stereolithography is limited; this is considered one of the major limitations of this technique^1^. For manufacturing biomedical scaffolds, the liquid resin also has to possess highly biocompatible properties; this often proves to be another system limitation based on existing photo-crosslinkable polymers.

The utilization of plant oils as feedstock for polymeric biomaterial synthesis is garnering greater attention^2^,^3^,^4^,^5^. As an important renewable resource, plant oils have been utilized to synthesize various polymers including polyesters, polyolefins, and polyurethanes^6^,^7^,^8^. In comparison to traditional biomaterials, plant oil polymers possess several desirable characteristics. Contrasted with petroleum-based biopolymers, which are from a limited and depleting resource, plant oil polymers are economical and renewable^9^. Plant oil polymers have also shown excellent biocompatibility. For example, phosphoester cross-linked vegetable oils and their metabolites have shown good cytocompatibility when tested on murine fibroblasts^3^. The polymer was completely degraded and absorbed by rats after a 3 month sub-dermal implantation eliciting a normal histological response^3^. Unlike other renewable polymers such as proteins and polysaccharides which have been widely used as biomaterials^10^,^11^, plant oil polymers are just emerging as suitable biomaterials for implantation. Fully exploring the use of plant oil polymers will provide a wide range of biomaterials which are valuable and complementary to existing natural biomaterials. To the best of our knowledge, studies on plant oil polymers as liquid resins for stereolithographical fabrication of biomedical scaffolds is rarely reported thus far.

The emerging technique of 4D printing refers to the ability of material objects to change form and function after they are 3D printed, offering additional capabilities and performance-driven applications^12^. For instance, water-expansible hydrophilic materials are 4D printed into self-evolving structures which perform geometric folding, curling, expansion and various other programmed shape changes after they are submerged in water^12^,^13^. 4D active composite materials are developed by printing shape memory polymer fibers in an elastomeric matrix achieving a programmed action through the stimulation of the shape memory fibers^14^,^15^. The time-dependent shape and/or functional changes realized with 4D fabrication techniques have shown great application potential for biomedical scaffolds^16^.

In this study, the key objective is to utilize soybean oil epoxidized acrylate as a liquid resin for fabricating 3D biomedical scaffolds and evaluate their biocompatibility with human bone marrow mesenchymal stem cells (hMSCs) which have great potential for various functional tissue applications (Fig. 1). To the best of our knowledge, we are the first to apply soybean oil epoxidized acrylate as an ink for fabricating biomedical scaffolds and evaluating their cytocompatibility. Furthermore, the fabricated scaffolds possess excellent shape memory effect, facilitating 4D functionality. This research significantly advances the development of biomedical scaffolds with renewable plant oils and 3D fabrication techniques.

## Results

3D printing is emerging tool for fabricating complex 3D scaffolds for tissue engineering. Here, we print biomedical scaffolds with soybean oil epoxidized acrylate using a novel, self-developed, table-top stereolithography printer which mirrors or outperforms commercial stereolithography systems^17^. The utilized ultraviolet (UV) laser is 355 nm. The effect of printing parameters, including print speed (from 10 to 80 mm/s) and laser frequency (from 8000 to 20000 Hz), on thickness and width of cured soybean oil epoxidized acrylate (Soy) is first investigated. As shown in Fig. 2A–D, the layer thickness decreases dramatically with the increase of print speed. When pint speed increases to 80 mm/s, the thickness is less than 100 μm which is roughly 22% of the thickness noted at the 10 mm/s print speed. The width also decreases with an increase in print speed; the width formed at a print speed of 80 mm/s is 250 μm, about 60% of the width generated at the 10 mm/s speed. On the contrary, the thickness and width slightly increase with increasing laser frequency ranging from 12000 to 20000 Hz. At a laser frequency of 8000 Hz, the thickness and width decreased dramatically to about 78% of the highest thickness and width.

Figure 2E shows the superficial SEM images of the fabricated scaffolds. The superficial structure of the scaffold is highly controlled by print speed and laser frequency. The scaffolds depicted in Fig. 2E(a–d) were constructed with a laser frequency of 20000 Hz at a print speed of 10 mm/s; the surfaces of these scaffolds are very coarse. Figure 2E(e–j) depicts scaffolds constructed with a laser frequency of 12000 Hz. For these scaffolds, the print speed varied from 10 to 80 mm/s. In Fig. 2E(k,l), the scaffolds were printed using a laser frequency of 16000 and 8000 Hz respectively with a speed of 10 mm/s. It is clear from these images that the struts in the scaffold become thinner when the laser frequency decreases and the fabricating speed increases. Figure 2F shows the resultant scaffolds which were printed with a laser frequency of 12000 Hz at a speed of 10 mm/s. When the infill density (represented in percentages indicating how much the resulting solid model is filled in with material) is 20–50%, visible pores are observed while no porous structure is noticed when the infill density is 70%. Considering that lower print speeds provide thicker and stronger struts, scaffolds printed with 10 mm/s at various laser frequencies were further analysed for this study.

**Figure 1:**
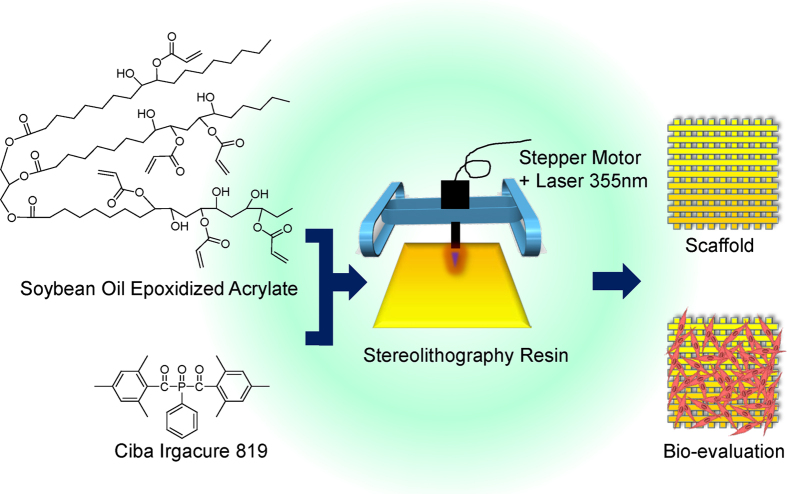
Schematic of soybean oil epoxidized acrylate fabrication process from raw material through resin fabrication and application.

**Figure 2:**
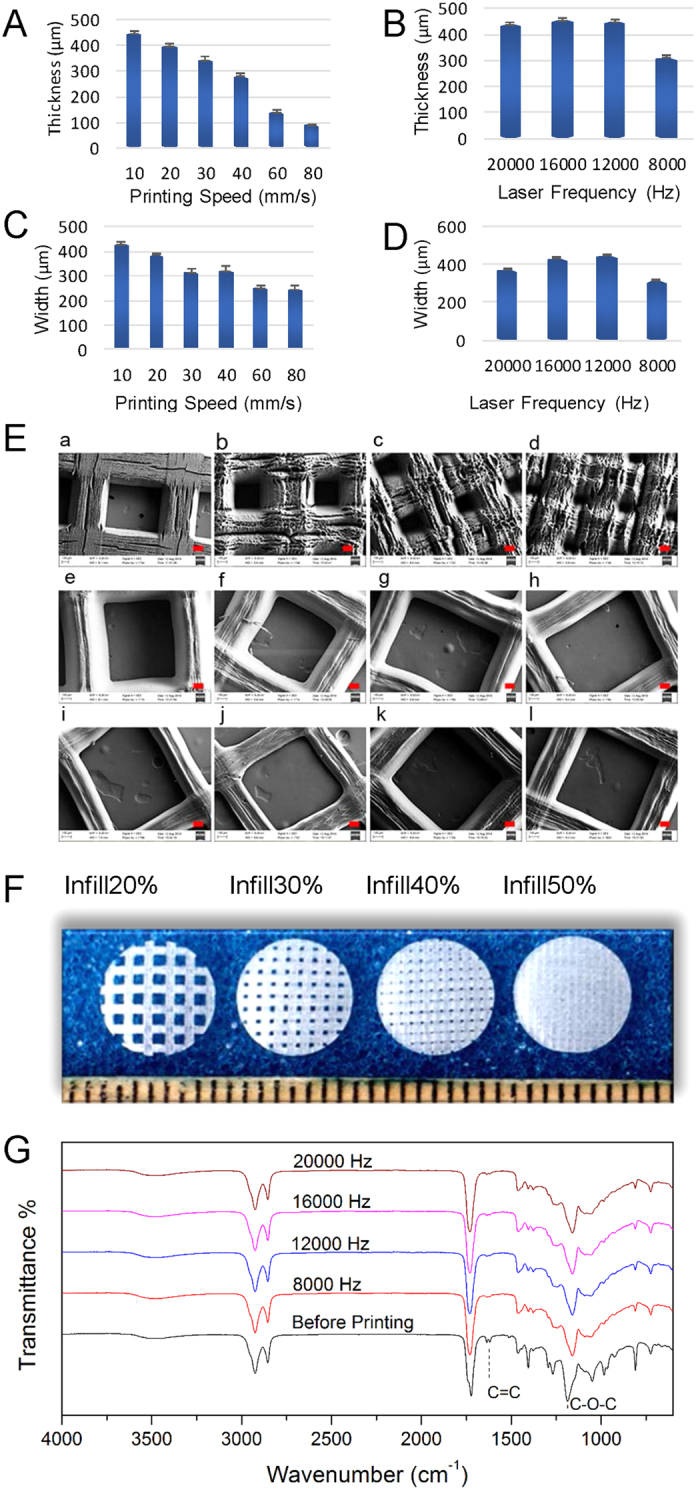
The effects of printing speed and laser frequency on printed scaffolds. (**A**) The effect of various printing speeds on thickness of struts at a laser frequency of 12000 Hz. (**B**) The effect of various laser frequencies on thickness of struts at a printing speed of 10 mm/s. (**C**) The effect of various printing speeds on width of struts at a laser frequency of 12000 Hz. (**D**) The effect of various laser frequencies on width of struts at a printing speed of 10 mm/s. (**E**) SEM images of printed scaffolds, red scale bar 100 μm. (a–d) Printing speed 10 mm/s, laser frequency 20000 Hz, infill density 20%, 30%, 40% and 50%, respectively; (e–j) Laser frequency 12000 Hz, infill density 20%, printing speeds 10, 20, 30, 40, 60, and 80 mm/s, respectively; (k,l) Infill density 20%, printing speed 10 mm/s, laser intensity 16000 and 8000 Hz, respectively. (**F**) The photos are of the printed scaffolds fabricated with laser frequency 12000 Hz and printing speed 10 mm/s with infill density 20%, 30%, 40% and 50% respectively. (**G**) FTIR analysis of soybean oil epoxidized acrylate and printed scaffolds with various laser frequencies at a printing speed of 10 mm/s. Data are mean ± standard deviation, n = 6.

FTIR analysis confirms the polymerization of soybean oil epoxidized acrylate, as shown in Fig. 2G. The signals from 1620 to 1640 cm^−1^ are corresponding to the C=C stretching in acrylic acid residues in soybean oil epoxidized acrylate. These signals decrease significantly after reaction, which indicates the polymerization of double bonds. The consumption of double bonds is further confirmed by the decrease of signals at 1407 and 810 cm^−1^ which are attributed to the oscillation of unsaturated double bonds. Another phenomenon is the signal shifts from 1186 cm^−1^ to 1177 cm^−1^ after reaction; this corresponds to the oscillation of C-O-C in ester group. As shown in Fig. 1, the C-O-C is next to the double bond; the consumption of double bonds has a great effect on the oscillation of C-O-C. No difference is noticed between the samples printed with different laser frequencies.

**Figure 3:**
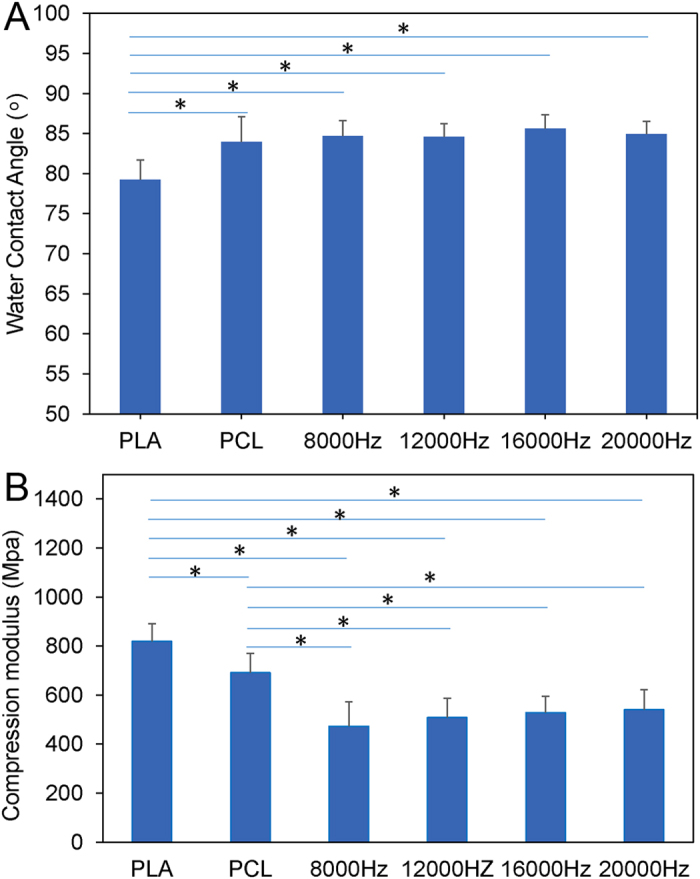
Water contact angle (**A**) and compression modulus (**B**) of the polymerized soybean oil epoxidized acrylate printed with various laser frequencies and infill density of 70% at a print speed of 10 mm/s compared with polylactic acid (PLA) and polycaprolactone (PCL). Data are mean ± standard deviation, n = 6. *p < 0.05.

The water contact angle of Soy is compared to poly lactic acid (PLA) and polycaprolactone (PCL), as shown in Fig. 3A. The water contact angle of Soy is significantly higher than that of PLA, but there is no statistical difference from PCL. No dramatic difference is noticed between the Soy samples printed with different laser frequencies. The compression modulus of Soy, compared to PLA and PCL, is shown in Fig. 3B. The compression modulus of Soy is lower than both PLA and PCL, and no dramatic difference is noticed between the Soy samples printed with different laser frequencies.

**Figure 4:**
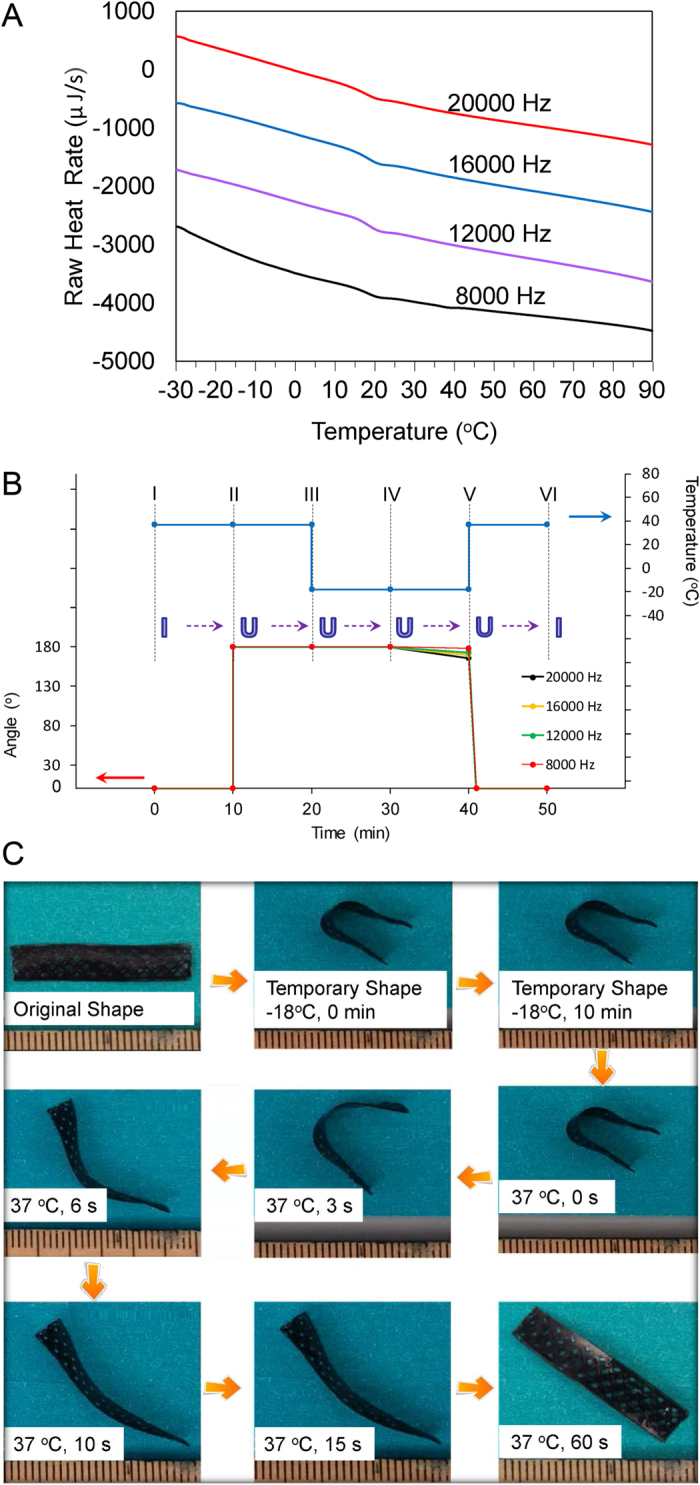
(**A**) DSC curves of the polymerized soybean oil epoxidized acrylate printed with a speed of 10 mm/s at various laser frequencies. (**B**) Shape memory circle of fabricated scaffolds at various laser frequencies: (I–II) The scaffold was kept at 37 °C for 10 min; (II–III) The scaffold was bent 180° and kept at 37 °C for 10 min; (III–IV) The bent scaffold was kept at −18 °C for 10 min; (IV–V) The external support was released and the scaffold was kept at −18 °C for another 10 min to determine the shape fixity; (V–VI) The scaffold was kept at 37 °C for 10 min to recover its original shape. (**C**) Shape memory circle demonstrated with a printed sample (laser frequency 12000 Hz, printing speed 10 mm/s) which was stained black to enhance the contrast with the background.

DSC analysis indicates that the Soy samples have a glass transition temperature (T_g_) of 20 °C (Fig. 4A). No melting peak is noticed, which indicates that the obtained polymer is highly cross-linked and has no crystalline domains. Therefore, Soy samples have two phases, glass and rubber, which are separated by the T_g_. In the glass phase (temperature <20 °C), the material is rigid and cannot be easily bent. On the other hand, when the temperature increases beyond T_g_ the material enters the soft rubber phase and its malleability increases. No difference is observed between Soy samples which are printed with various laser frequencies.

The printed Soy scaffolds also display temperature sensitive shape memory effect. As shown in Fig. 4B, the sample is bent into a U shape at 37 °C, and kept at this temperature for 10 min (II–III). Next the temperature is reduced to −18 °C and the sample is maintained at this temperature for 10min (III–IV). The external mechanical force, which is applied to restrict the U shape, is then removed (IV–V) revealing a fixed, temporary shape for all of the samples. The sample printed with 8000 Hz has the highest shape fixity of 99%; the sample printed with 20000 Hz exhibits the lowest shape fixity of 92%. When the samples are placed in 37 °C (V–VI), all fully recover their initial and permanent shape within 1 min. The shape memory process is demonstrated in Fig. 4C. This further confirms that the printed Soy samples have excellent shape memory effect.

**Figure 5:**
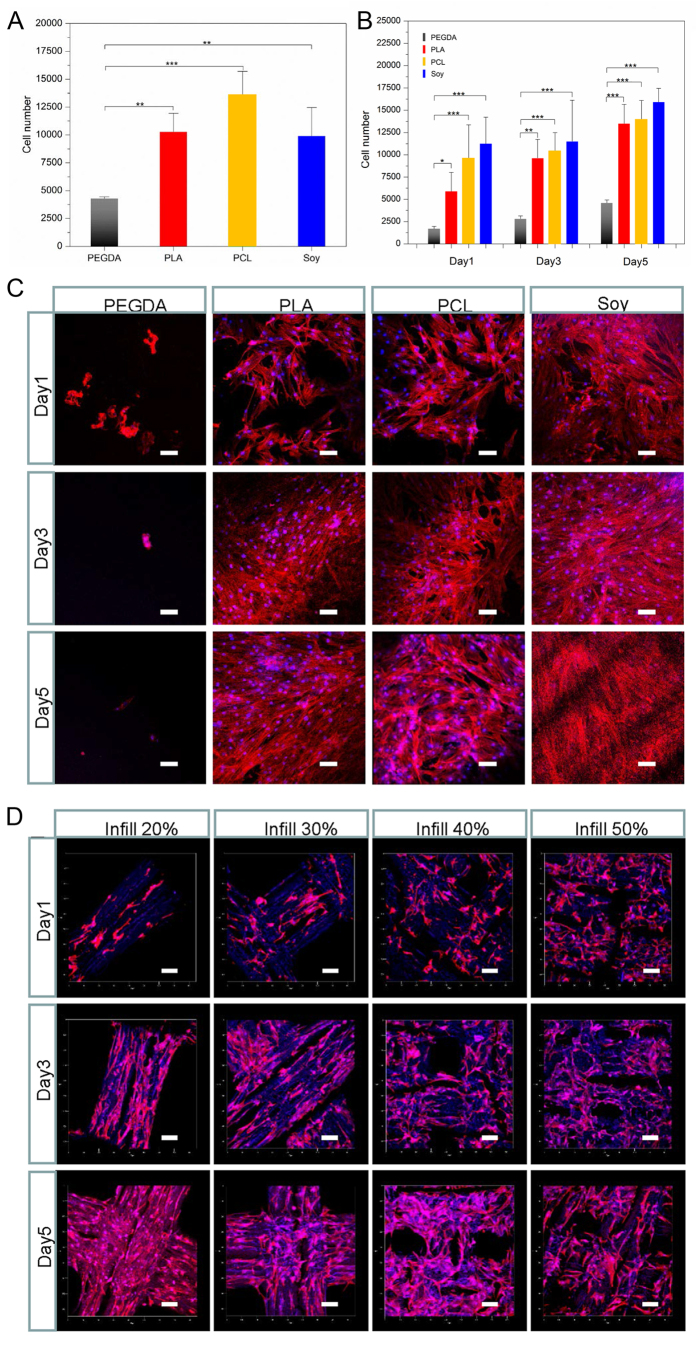
hMSC attachment (**A**) and proliferation (**B**) on PEGDA, PLA, PCL and Soy. Confocal images of hMSCs spreading on different materials (**C**) and printed scaffolds from soybean oil epoxidized acrylate (printing speed 10 mm/s, laser frequency 20000 Hz) with different infill density (**D**). Data are mean ± standard deviation, n = 6. *p < 0.05, **p < 0.01, ***p < 0.001. Scale bars are 100 μm.

The attachment of hMSCs on Soy samples is further evaluated and compared to polyethylene glycol diacrylate (PEGDA), PLA and PCL. The Soy sample is printed with 70% infill, 12000 Hz frequency, and a speed of 10 mm/s. As shown in Fig. 5A, the Soy sample has significantly higher attachment capability than PEGDA, but is not statistically different from PLA and PCL. The hMSC proliferation results are shown in Fig. 5B. Similarly, PEGDA has the lowest hMSC proliferation potential while there is no statistical difference among the other samples. The hMSC performance is further observed on the different materials with confocal analysis; the results are shown in Fig. 5C. PEGDA is almost void of cells. In contrast, all other materials support excellent attachment and spreading. Again, there is no noticeable difference among Soy, PLA and PCL groups. Figure 5D displays the cell spreading on the surfaces with varied infill patterns as shown in Fig. 2E(a–d). All the samples display favourable hMSC proliferation. It appears that the hMSCs tend to grow along the wrinkled structures on the surface, especially with the 20% infill; this may indicate a novel method for cell alignment but is beyond the range of this study.

## Discussion

Soybean oil epoxidized acrylate is a novel liquid resin for biocompatible scaffold fabrication using multi-dimensional stereolithography. PEGDA is one of the most commonly investigated resins for biomedical scaffolds thus far, in spite of well known challenges due to its inherent bio inert properties. Nearly all types of cells cannot effectively adhere and grow on its surface^18^. PLA and PCL are highly biocompatible polymers, but they can’t be directly used as resins for stereolithography because of their lack of photo sensitive chemical groups. Great efforts have been made to synthesize PLA and/or PCL based liquid resin for stereolithography. The macromers based on trimethylene carbonate and caprolactone oligomers are utilized to develop three-dimensional cartilage regeneration scaffolds by microstereolithography^19^. PLA oligomers end-functionalized with an unsaturated moiety such as a methacrylate-, acrylate-, and fumarate- groups are mostly utilized to develop photo-curable resin^20^,^21^,^22^,^23^. However, these macromers need to be heated or diluted with reactive diluents such as methyl methacrylate and butane-dimethacrylate to make liquid resin^24^,^25^. In comparison to these liquids, soybean oil epoxidized acrylate possesses advantages on both processing and biocompatibility. Soybean oil epoxidized acrylate is liquid at room temperature, and doesn’t need any heating and/or reactive diluents for stereolithography. The printed scaffold in this study has significantly higher attachment and proliferation of hMSCs than PEGDA, and performs on par with PLA and PCL. The excellent cytocompatibility of the Soy samples may be related to contact angle or chemical groups, among other reasons. The water contact angle for the Soy samples closely resembles that of PCL which might partially contribute to good cell attachment and growth comparable with the excellent biocompatibility of PCL (Fig. 3A). Also, in the Soy resin there are only two types of chemical groups, hydroxyl and ester (Fig. 1); both groups are mostly cyto-benign.

Laser frequency affects struts’ thickness and width, but no difference is observed between the Soy samples printed with various laser frequencies in the FTIR, water contact angle, compression modulus and DSC analyses. This may be due to the soaking of the printed samples in 95% ethanol to remove unreacted soybean oil epoxidized acrylate after printing; the remaining polymerized soybean oil epoxidized acrylate may have too slight of a difference to be detected.

**Figure 6:**
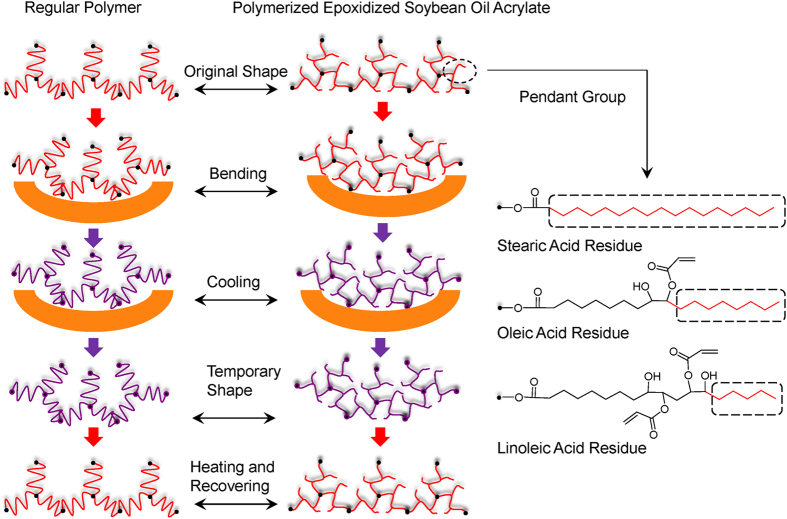
Schematic illustration of the difference between polymerized soybean oil epoxidized acrylate and regular polymer on shape memory mechanism.

The printed scaffolds have excellent shape memory effect, which is dependent on the T_g_ of the polymerized soybean oil epoxidized acrylate. The high shape fixity and recovery are attributed to the chemical cross-linking induced by laser printing. It has been reported that covalently cross-linked polymers generally exhibit superior properties compared with thermoplastic elastomers^26^. The shape memory circle of regular T_g_-based polymers is illustrated in Fig. 6. When the temperature is lower than its T_g_, the linear segments between cross-linking points are frozen, which fixes a temporary shape. The sample will recover its original shape, attributed to the chemical cross-linking, after the temperature is increased to greater than the T_g_. For the polymerized soybean oil epoxidized acrylate, the specific pendant groups may also play an important role in shape memory effect. As shown in Fig. 6, in soybean oil there are three major fatty acid residues: stearic, oleic and linoleic acids, which have pendant alkane groups. At −18 °C, these groups may freeze benefitting the shape fixity; at 37 °C, the oscillation of these groups may contribute to the full shape recovery. The function of the pendant groups is also observed in soybean oil-based polyurethanes^27^,^28^.

Bio-based chemicals are getting increased attention as liquid resins for stereolithography of biomedical scaffolds. Most of the macromers for stereolithography are petroleum-based, although bio-based biomedical polymers have received increasing attention in recent years^2^. PLA and PCL can be produced from renewable resources, but modification of PLA and PCL to form photo-curable resins is tedious and involves toxic chemicals, as mentioned earlier. Plant oils have unsaturated double bonds which are easily reactive and induce functional groups including epoxy and other more active double bonds^2^. The utilization of soybean epoxidized acrylate in this study inspires the exploration of other plant oils and bio-based chemicals as liquid resins for constructing biomedical scaffolds.

4D fabrication is receiving increasing attention in the past two years. With 4D printing, the fabricated object can change their shape and/or function on-demand and over time, which may have great potential for developing scaffolds that only become active when encounter particular environments in the body^16^,^29^. The two 4D approaches investigated the most thus far are designing multi material architectures and using functional materials. However, functional resin is extremely limited in its ability to achieve a 4D self-assembly construct. Shape memory polymers show great potential for additional morphological changes achieving the desirable 4D effect, but liquid resins for fabricating shape memory scaffolds via stereolithography is rarely reported. In this study, soybean epoxidized acrylate based shape memory scaffolds were easily obtained. This significantly advances the investigation of various compositions from plant oils and other renewable chemicals to achieve advanced 4D fabrication techniques.

## Conclusions

Soybean oil epoxidized acrylate is a novel and renewable liquid resin for multi-dimensional stereolithography of biomedical scaffolds. Soybean oil epoxidized acrylate is readily polymerizable by ultraviolet laser, and the solidified resin possesses excellent shape memory effects, which has great potential for additional 4D effects. The fabricated scaffold is highly biocompatible with significantly higher attachment and proliferation of hMSCs compared to PEGDA, and has no statistical difference from PLA and PCL which are highly biocompatible and clinically approved biomaterials. This research will significantly advance the utilization of renewable resources for constructing biomedical scaffolds using the stereolithography printing technique; additionally the contribution of this material toward realizing novel, advanced 4D constructs provides additional significance.

## Methods

### Preparation of 3D printing ink

100 g soybean oil epoxidized acrylate (contains 4,000 ppm monomethyl ether hydroquinone as inhibitor, Sigma-Aldrich, USA; used as received) was mixed with 100 mL acetone. Then 1.26 g bis(2,4,6-trimethylbenzoyl)-phenylphosphineoxide (Ciba Irgacure 819) (Ciba Specialty Chemicals, Switzerland) was added. The mixture was shaken mildly to get a homogenous yellow solution which was subsequently put into a vacuumed container overnight to remove acetone. The obtained yellow liquid was used as ink for 3D printing.

### 3D Printing of biomedical scaffolds

Scaffolds were printed via stereolithography with a table top SL system developed in our lab, based on the existing Solidoodle® 3D printer platform^17^. Open source software (Prontrface®) was employed to control the 3 stepper motors with an effective resolution of 100 μm in x, y, and z-axis. The major modification to the existing platform is the incorporation of a 110 μm fiber optic-coupled solid-state UV (355 nm) laser (MarketTech, Scotts Valley, CA). Per the manufacturer’s specifications, the effective spot size of the emitted light is 190 ± 50 μm with an energy output of ∼20 μJ at 15 kHz. A glass petri dish fixed on the print bed acted as a minivat for the addition of liquid photocurable resin. The ability to alter the frequency of the pulsed signal facilitates power control at the material’s surface ranging ∼40–110 mW. The ink was cured by activating the laser and drawing lines at various print speeds. After polymerization, the scaffold was lifted off the petri dish and was soaked in 95% ethanol for overnight to remove unpolymerized ink and photo initiator. Then the scaffold was sterilized with 70% ethanol for 30 min and soaked in phosphate buffered saline (PBS) overnight prior to cell culturing.

### Characterization

A Fourier transform infrared spectroscopy spectrometer (Nicolet Series II Magna-IR System 750, Nicolet Instrument Inc.) equipped with a horizontal germanium attenuated total reflectance accessory (ATR-FTIR) was used to evaluate samples. The scan range used was 600 to 4000 cm^−1^ with a resolution of 4 cm^−1^. Surface topography analysis of the synthesized polymers was performed via a focused ion beam operating in scanning electron microscopy (SEM) mode (Zeiss NVision 40 FIB) under an accelerating voltage of 1–2 kV. All samples for SEM were sputter-coated with gold. Surface wettability of test specimens was measured using a contact angle analyzer (DSA4; Krüss). Approximately 3 μL of ultrapure H_2_O was deposited on the samples’ surface and recorded. Static water contact angle measurements were obtained from the first image of every recording. All experiments were conducted in ambient conditions and repeated five times per sample. Uniaxial compression tests were conducted using a uniaxial mechanical tester from MTS Systems Corporation (Eden Prairie, MN). A flat 2 cm diameter platen attached to a 100 N load cell was advanced upon the sample (8 mm diameter cylinder, 2 mm high) at a test speed of 10 mm/min and strain endpoint of 5 mm/mm. Data were taken using LabView software (National Instruments Corporation, Austin, TX) and Young’s modulus were determined by the linear elastic region. The T_g_s of the synthesized polymers were measured with a multi-cell differential scanning calorimeter (MC DSC) from TA Instruments (New Castle, DE) at a programmed ramp rate of 1 °C/min. The sample was first heated from 25 to 150 °C, and held at 150 °C for 1 min. Then the sample was cooled from 150 to −30 °C, and held at −30 °C for 1 min. A second cycle was conducted: heating from −30 to 150 °C, holding 1 min and decreasing from 150 to −30 °C. The second cycle was used to determine the T_g_s. Shape memory tests were conducted according to a reported method with slight modification^30^. The scaffold was printed into 75 × 10 mm strips with an infill density of 30%. The edges of the strips were stained with black dye for increased optical contrast. The strips were folded 180° at 37 °C into a “U” shape using a mold possessing an inner radius of 10 mm, and kept at this temperature for 10 min. The samples were then immediately cooled to a preset temperature (−18 °C) and maintained at this temperature for an additional 10 min. The mold was removed and the test strips were kept at the −18 °C preset temperature for another 10 min. The fixed angle of the specimen was determined and recorded as θ_fixed_. The strips were then immersed in 37 °C PBS immediately to recover the permanent shape. The final angle of the specimen was determined and recorded as θ_final_. Shape fixity (R_f_) and shape recovery (R_r_) were calculated by the following equations:


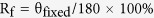



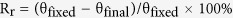


### Cytotoxicity evaluation

Primary human bone marrow mesenchymal stem cells (hMSCs) were obtained from healthy consenting donors at the Texas A&M Health Science Center, Institute for Regenerative Medicine. hMSCs (passage No. 3–6) were cultured in complete media composed of alpha minimum essential medium (Gibco) supplemented with 16.5% fetal bovine serum (FBS) (Atlanta Biologicals), 1% (v/v) l-glutamine (Invitrogen), and 1% penicillin:streptomycin solution (Invitrogen) and cultured under standard cell culture conditions (37 °C, a humidified, 5% CO_2_/95% air environment). For hMSC attachment studies, the polymer test samples were cut into 8 mm diameter specimens. hMSCs were seeded at a cell density of 50,000 cells/specimen, and cultured under standard cell culture conditions for 4 h. The specimens were washed three times with PBS to remove non-adherent cells. The adhered cells were lifted with trypsin-ethylenediaminetetraacetic acid and quantified with CellTiter 96″ Aqueous Non-Radioactive Cell Proliferation Assay and analyzed spectrophotometrically using a Thermo Scientific Multiskan GO Spectrophotometer at 490 nm. For proliferation studies, hMSCs were seeded at a density of 10,000 cells/scaffold and cultured for 1, 3, and 5 days, respectively. Media was exchanged every other day and cells were lifted for analysis via MTS assay as previously described. Furthermore, confocal microscopy was used to characterize hMSC growth and spreading morphology for 1, 3, and 5 days. At each time point, samples were washed twice with PBS, fixed with 10% formalin and permeabilized in 0.1% Triton X-100. After rinsing with PBS, the remaining cells were stained with Texas red fluorescent dye (to stain the cells’ cytoskeleton) for 30 min and then DAPI blue fluorescent dye (to stain the cells’ nuclei) for 15 min. The double-stained samples were imaged on a Zeiss LSM 710 confocal microscope.

### Statistical Analysis

Statistics for quantitative tests were performed using ANOVA and Tukey’s multiple pairwise comparison (p < 0.05 for significance) unless otherwise stated. Values reported are mean ± standard deviation, and significant differences are specified in figures.

## Additional Information

**How to cite this article**: Miao, S. *et al*. 4D printing smart biomedical scaffolds with novel soybean oil epoxidized acrylate. *Sci. Rep.* **6**, 27226; doi: 10.1038/srep27226 (2016).
